# Bifurcated Asymmetric Field Flow Fractionation of Nanoparticles in PDMS-Free Microfluidic Devices for Applications in Label-Free Extracellular Vesicle Separation

**DOI:** 10.3390/polym15040789

**Published:** 2023-02-04

**Authors:** Miks Priedols, Gunita Paidere, Cristina Bajo Santos, Antons Miscenko, Romualds Gerulis Bergmanis, Arnita Spule, Beate Bekere, Gatis Mozolevskis, Arturs Abols, Roberts Rimsa

**Affiliations:** 1 Latvian Biomedical Research and Study Centre, Ratsupites Str. 1, k-1, LV-1067 Riga, Latvia; 2 Institute of Solid-State Physics, University of Latvia, 8 Kengaraga Str., LV-1063 Riga, Latvia; 3 Cellbox Labs LLC, 8 Kengaraga Str., LV-1063 Riga, Latvia; 4 Faculty of Materials Science and Applied Chemistry, Riga Technical University, LV-1048 Riga, Latvia

**Keywords:** extracellular vesicles, microfluidics, field flow fractionation, nanoparticles, PDMS, OSTE

## Abstract

Extracellular vesicles are small membrane-bound structures that are released by cells and play important roles in intercellular communication garnering significant attention in scientific society recently due to their potential as diagnostic and therapeutic tools. However, separating EVs from large-volume samples remains a challenge due to their small size and low concentration. In this manuscript, we presented a novel method for separating polystyrene beads as control and extracellular vesicles from large sample volumes using bifurcated asymmetric field flow fractionation in PDMS-free microfluidic devices. Separation characteristics were evaluated using the control system of polystyrene bead mix, which offers up to 3.7X enrichment of EV-sized beads. Furthermore, in the EV-sample from bioreactor culture media, we observed a notable population distribution shift of extracellular vesicles. Herein presented novel PDMS-free microfluidic device fabrication protocol resulted in devices with reduced EV-loss compared to size-exclusion columns. This method represented an improvement over the current state of the art in terms of EV separation from large sample volumes through the use of novel field flow fractionation design.

## 1. Introduction

Extracellular vesicles (EVs) are bi-lipid encapsulated particles of microscopic size released by any cell type to the extracellular space [1]. Based on their biogenesis, they are originated either by invagination of the plasma membrane and release via exocytosis; or intracellularly by inward budding of the endosomal membrane in the vesicular body, which later fuse with the plasma membrane, followed by secretion to the extracellular environment [2,3]. EVs have been shown to play a crucial role in cell communication processes regulating pathological and physiological conditions through the specific transfer and sorting of their mediated cargo. Due to this, EVs have arisen as promising novel biomarkers and therapeutic targets to treat and diagnose several diseases, including cancer [1,4].

In order to utilize EVs as a diagnostic and therapeutic tool, EVs need to be isolated from biofluids and cell media, respectively. The main isolation techniques currently used are ultracentrifugation (UC), size-exclusion-chromatography (SEC), density gradient centrifugation, precipitation, and immunoaffinity purification. These techniques rely on the different buoyant densities, size selection, precipitation rates, and affinity interactions of different EV markers [5]. All methods differ in purity, recovery yield, and efficiency, leading to poor reproducibility [6]. Microfluidic devices hold great promise to solve this problem due to simplified workflows, short operation times, superior small sample size handling, and excellent fabrication repeatability [7,8]. Indeed, several microfluidic-based solutions for EV isolation have been published; however, the majority of these techniques are only optimal for small volume samples (up to 1 mL), which either requires concentrating the sample beforehand that can cause EV aggregation or using filtration, that limits sample volume even more since filters can clog up and decrease EV yield [9]. This is not suitable for large-volume samples (>1 mL), such as cell media from bioreactors to produce EVs for therapeutic purposes or urine samples to utilize EVs as non-invasive diagnostic markers for different diseases. Additionally, in the field of EV research utilizing microfluidics, polydimethylsiloxane (PDMS) material remains the dominant material of choice for device fabrication due to simple fabrication and bonding processes and the plethora of various fabrication protocols [10,11]. However, it is well known that PDMS suffers from high lipophilic molecule absorption [12,13], which potentially can cause issues with its applications in the EV field, given the structure of the EVs. Additionally, PDMS is not suitable for mass manufacturing, which can increase the variability of sample isolation due to the device fabrication methods. Cyclic olefin copolymer (COC) has many advantages as the substrate of the microfluidic chips, such as biocompatibility, high chemical resistance, and small non-specific absorption as well as compatibility with large scale manufacturing, yet it is complex to assemble devices without specialized equipment [14]. Off-stoichiometry thiol-ene (OSTE) has been shown as a promising alternative to PDMS given the significantly reduced small molecule absorption yet retaining the ease of fabrication [15,16]. Therefore, we have combined the ease of bonding of OSTE and the superior properties of COC into a single fabrication process [17] to create improved asymmetrical flow field-flow fractionation (A4F) devices for EV isolation.

A4F is a label-free entity separation method that utilizes a porous membrane in the floor of a microfluidic channel, thus creating a downward force known as cross-flow onto particles traveling in the channels. A typical workflow sample is injected in the channel system and focused through the inlet and outlet flows to generate the separation of the entities prior to running the sample in the microfluidic channels, where entities are laterally size-separated due to Poiseuille flow [18]. Examples of separated entities include both micro- and nano-sized entities, such as nanoparticles [19], extracellular vesicles [20], and others [21]. However, due to these loading and focusing steps, the total loadable sample size is small; therefore, a significant amount of research in multiplexing [22] and size-scaling [23] has been done. However, none of these methods currently address the fact that this separation cannot be done continuously, which is necessary for particularly large sample volumes.

Herein, we presented a novel microfluidic device developed from an alternative to PDMS polymers that utilizes the continuous flow bifurcated A4F method to separate EVs from cell debris, which improves the currently existing A4F method through improved confinement of particles closer to the membrane, thus allowing continuous flow operation. The system is characterized by using fluorescent particles as a proof of concept for EV separation prior to showing EV-retention experiments with standard-characterized EVs. Finally, the presented device uses a novel PDMS-free fabrication protocol that could allow using this device for mass manufacturing in the future through the reaction injection molding process [24].

## 2. Materials and Methods

### 2.1. Cell Culturing in Bioreactor and Extracellular Vesicle Isolation

The hTERT human immortalized adipose-derived Mesenchymal stem cells (ASC52telo) used for EV production were purchased from the American Type Culture Collection (ATCC, Manassas, VA, USA). Cells were cultured in DMEM medium supplemented with 2% Fetal Bovine Serum (Sigma-Aldrich, St. Louis, MO, USA, #F7524), 1.2% GlutaMAX (ThermoFisher, A1286001), 10 ng/mL basic fibroblast growth factor (βFGF) (Santa Cruz, Biotechnology, Dallas, TX, USA, #sc- 4573), 5 ng/mL epidermal growth factor (EGF) (R&D Systems, Minneapolis, MN, USA, #236-EG-200), 100 μg/mL Primocin^®^ (Invivogen, San Diego, CA, USA, #ant-pm-2) & 200 μg/mL antibiotic G418 (Thermo Fisher Scientific, Waltham, MA, USA, #11811-023). All cells were cultured in humidified 5% CO_2_ incubator at 37 °C incubator.

After expansion, 180 million ASC52telo cells were seeded in a hollow-fiber bioreactor (Fibercell Systems, New Market, MD, USA, #C2011) extracapillary space (ECS) of a culture cartridge containing densely packed hollow fibers. Nutrients/waste could move freely across the hollow-fiber membrane (20 kDa MWCO), while large cellular products (including EV) accumulated in the ECS. To monitor cell viability, daily glucose measurements were taken. Once glucose consumption had reached 1000 mg/24 h, the medium was gradually replaced with a serum-free medium containing Chemically Defined Medium-High Density (CDM-HD) (Fibercell systems, #CDM-HD). 20 mL of ECS was harvested every day. Media was centrifuged at 300× *g* for 5 min at room temperature to remove cells and at 3000× *g* for 30 min to remove cell debris before storing at +4° C for further EV isolation. Additionally, once a week, the ECS was vigorously washed with cell media to remove dead cells and cell debris with the intention of promoting new cell growth.

EV isolation was performed every four days, where four ECS media collections were pooled together for a single batch of EVs. Then, media was concentrated up to 5 mL using 100 kDa centrifuge filters (Merck Millipore, MA, USA, #UFC910024) at 3000× *g* for 2 h at +4 °C. Size exclusion chromatography (SEC) was performed to separate EVs from protein aggregates by using Izon qEV10/35 nm columns (Izon, Christchurch, New Zealand #SP7). Further, each SEC fraction was measured by Zetasizer Nano ZS (Malvern panalytical, Malvern, UK), and all fractions containing particles with mean size bigger than 30 nm were combined and concentrated up to 250 μL using 3 kDa filters (Merck Millipore, #UFC500324) at 14,000× *g* for 2 h at +4 C. Finally, the concentrates were aliquoted and frozen at −80 °C to avert multiple freeze/thaw cycles.

### 2.2. Extracellular Vesicle Characterization

EV characterization was performed by Nanoparticle Tracking Analysis (NTA), Western Blotting (WB), double sandwich enzyme-linked immunoassay (dsELISA), and transmission electron microscopy (TEM) according to MISEV2018 guidelines [25].

Size, distribution profile, and concentration of EVs were determined using NTA NanoSight NS300 instrument (Malvern, UK) equipped with green (532 nm) laser and complementary scientific metal–oxide–semiconductor (sCMOS) camera. EV samples were diluted usually from 200 to 500-fold in 0.02 µL filtered phosphate-buffered saline (PBS, Fisher Scientific, BP2944-100) to achieve particle concentration in the range of 7 × 10^7^ to 7 × 10^8^ particles/mL, which is the optimal working range for NTA. For each sample, five 60 s videos were recorded with the following settings: 25 °C, 0.944–0.948 cP, 1000 slider shutter, 400 slider gain, and camera level 14. Data analysis was performed with NanoSight NTA Software v3.4 Build 3.4.003 with detection threshold 9.

EV marker expression was assessed using dsELISA or WB. DsELISA was performed as previously described [26]. A 96-well ELISA plate (ThermoFisher Scientific, Wilmongton, DE, USA) was coated with 1 µg/ul TIM4-Fc (Adipogen LifeSciences, SanDiego, CA, USA) and incubated overnight at 4 °C. Next morning, wells were blocked with 1% BSA diluted in 0.05% TBST, followed by inoculation of 1/100 of EV sample diluted in 0.05% TBST supplemented with 20 mM CaCl_2_. Samples were incubated with 1:300 CD63 (SantaCruz, SC-5275) as the primary antibody followed by 1:1000 anti-mouse secondary antibody (Santa Cruz, sc-516102). The reaction was activated through the addition of TMB reagent, and after 30 min, the reaction was stopped using 1 M H_2_SO_4_ solution. The plate was read in a spectrophotometer at 450 nm. Data was obtained using Gen5 software. Samples were analyzed in duplicates.

In order to perform WB analysis, proteins were extracted from EVs with Radioimmunoprecipitation Assay buffer (RIPA) (50 mM Tris, pH 8.0, 0.6 M NaCl, 4% Triton X-100, 2% sodium deoxycholate, 0.1% SDS). The concentration of acquired proteins was measured using a Pierce BCA Protein assay kit (ThermoFisher Scientific, Wilmington, DE, USA) following the manufacturer’s instructions and normalized as a starting input. The protein amount of 10 μg of EV total protein was loaded per lane and separated by 10% SDS-PAGE. Proteins were then electroblotted upon nitrocellulose membranes, blocked with 10% (*w*/*v*) fat-free milk, and then incubated with the following primary antibodies: TSG101 (Abcam, ab125011) (1:1000) and CD63 (SantaCruz, SC-5275) (1:500). The membranes were washed and incubated with peroxidase-conjugated rabbit anti-mouse secondary antibody (Santa Cruz, sc-516102) (1:2000) or mouse anti-rabbit secondary antibody (Santa Cruz, sc-2357) (1:2000). Then, membranes were visualized using ECL Select Western Blotting Detection Reagents (GE Healthcare, RP2235) according to the manufacturer’s instructions. TEM was performed as described before [27]. Briefly, EV samples (10 μL) were fixated for 5 min in a 300-mesh carbon-coated copper EM grid followed by 1-min incubation with 1% uranyl format (*w*/*v*). Samples were visualized using a JEM-1230 transmission electron microscope (TEM) (JEOL, USA).

For experiments, EV aliquots were slowly thawed on ice, vortexed, and spun. Samples were diluted in 0.02 µm filtered PBS to achieve a calculated concentration necessary for each experiment; however, dilutions before experiments were measured again with NTA as described above to quantify the exact number of EVs.

### 2.3. Single Channel PDMS Device Fabrication

**Figure 1:**
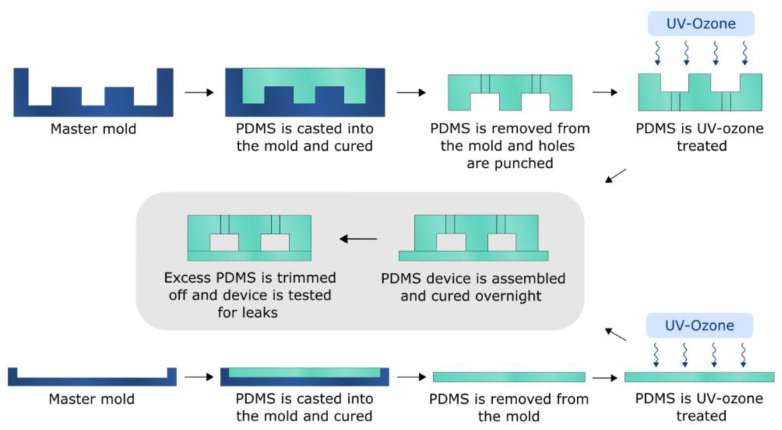
PDMS single-channel EV separation device fabrication workflow.

For device fabrication, custom 3D printed molds were designed in SOLIDWORKS 2020 (Dassault Systèmes, France) computer-aided design (CAD) software and fabricated using an ultraviolet light liquid crystal display (UV LCD) 3D printer (Zortrax Inkspire, Olsztyn, Poland). The molds were pretreated according to the protocol mentioned by Venzac et al. [28]. Single-channel PDMS devices were fabricated by casting PDMS (Sylgard 184, Dow Corning, Midland, MI, USA, 1:10 crosslinker/base ratio, weight/weight (*w*/*w*)) onto a custom 3D printed mold with channel height of 0.5 mm, width of 1.5 mm, and length of 27 mm. In another round-shaped mold with a diameter of 85 mm and height of 13 mm, a 2 mm PDMS layer was cast. Degassing of PDMS was done at –800 mbar pressure for about 30 min. After curing overnight at 60 °C, PDMS was removed from the molds, and 1.2 mm holes were punched in the PDMS-containing channels using a biopsy puncher. The PDMS parts were bonded by UV-ozone exposure (Novascan PSDP-UV8T, Boone, USA) for 7 min and cured overnight at 60 °C with an applied pressure of around 2 kPa, as visualized in Figure 1. After curing, excess PDMS was trimmed off, and the device was tested for leaks. To evaluate the devices’ bonding performance, deionized water (DIW) filtered with a 0.02 µm syringe filter was passed through each channel using a pressure system with a 30 mbar pressure for 1 min and 100 mbar pressure for ~10 s for each channel. During the pressure testing, the device was carefully examined for leaks. Devices that passed this step were used further for EV recovery and adsorption tests.

### 2.4. Single Channel OSTE-COC Device Fabrication

**Figure 2:**
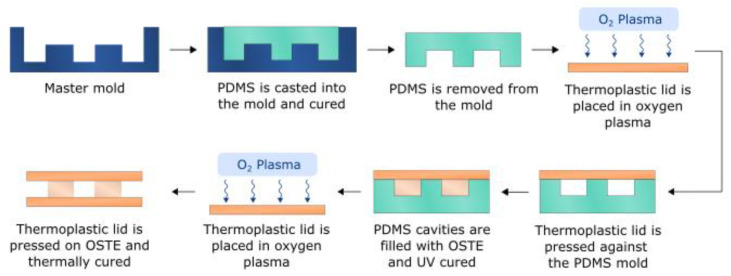
OSTE-COC single-channel EV separation device fabrication workflow.

To fabricate single-channel OSTE-COC devices, the mold was formed by casting PDMS (QSIL 216, PPS, USA, 1:10 crosslinker/base ratio, *w*/*w*) in a custom 3D-printed master mold with the same channel dimensions as for PDMS devices described previously. Degassing of PDMS was done at –800 mbar pressure for about 30 min. To ensure that the PDMS molds are the same height, a 100 µm PVC film is carefully placed over liquid PDMS as described in [29], and an acrylic lid was placed on top. After curing overnight at 60 °C, the mold was removed from the master mold, and 0.5 mm holes were punched using a biopsy puncher. OSTE 322 (Mercene Labs, Stockholm, Sweden) was mixed as per instructions on the bottle (1.09:1 Part A/Part B, *w*/*w*) and degassed for 30 min at −800 mbar pressure. A clean COC slide with mini luer connections (microfluidic ChipShop, Jena, Germany) was O_2_ plasma treated (PVA TePla AG GIGAbatch 360 M, Wettenberg, Germany) for 2 min and pressed against the PDMS mold. Subsequently, OSTE was filled into the mold cavity using a pressure system (Elveflow OB1 MK3+, Darwin microfluidics, Paris, France) and cured with a UV dose (Mask aligner Suss MA/BA6, Suss Microtec, Garching, Germany) of 750 mJ/cm^2^. After the UV curing step, the OSTE surface was brought in contact with an O_2_ plasma-treated clean COC slide (microfluidic ChipShop, Jena, Germany), as shown in Figure 2, and cured overnight at 60 °C with an applied pressure of ~1.6 kPa. Bonding performance tests were performed using the same method as for the single-channel PDMS devices.

### 2.5. Microfluidic Setup for Single Channel Devices

**Figure 3:**
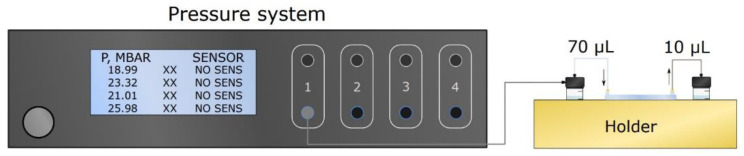
Pressure system setup used to provide fluid flow for single channel PDMS and OSTE-COC devices.

To ensure fluid flow in the single channel devices, a pressure system (Elveflow OB1 MK3+, Darwin microfluidics, Paris, France) set-up was used, as shown in Figure 3. Polytetrafluoroethylene (PTFE) tubing (800 µm inner diameter (ID), Darwin microfluidics, Paris, France) was connected to inlets, whilst polyether ether ketone (PEEK) tubing (250 µm ID, Darwin microfluidics, Paris, France) were connected to the outlets to ensure uniform resistance at channel outlets. To precisely determine flow rates depending on the pressure applied, calibration tests were performed. Pressure values used for calibration—50 mbar, 100 mbar, and 150 mbar—corresponded with flow rates of 222 µL/min, 544 µL/min, and 866 µL/min, respectively. The use of a pressure system allowed us to keep utilizing low-retention centrifuge tubing for EV-sample handling, thus paving the way for accurate EV-loss quantification in single-channel devices.

### 2.6. PDMS and OSTE-COC Single Channel EV Recovery and Adsorption Comparison

First, each channel was washed with 1 mL of 0.02 µm filtered 3% H_2_O_2_, 1 mL of 0.02 µm filtered 70% ethanol, and 4 mL of 0.02 µm filtered PBS to clean the microfluidic channels according to the in-house developed protocol, followed by overnight incubation with PBS to collect leaked polymer particles. The next day all channels were washed again with 4 mL of 0.02 µm filtered PBS to remove any particles that could have leaked into the channel from the device itself. Afterward, a blank was made by running 1 mL of 0.02 µm filtered PBS through each channel and analyzed using Nanosight NS300, similar to EVs as described in the Section 2.2. The channel was then purged with compressed, filtered air, and the EV sample was ready to be run.

EV sample dilution was prepared as described in Section 2.2 before the experiment from 3.5 × 10^8^ to 5.8 × 10^8^ particles/mL according to NTA data. Next, samples were run through each channel using 100 mBar pressure, collected, and analyzed using Nanosight NS300. The EV samples, without running through the device, were used as a reference after dilution. In total, 7 biological replicates were performed. EV recovery percent was calculated using sample concentration data obtained using Nanosight NS300 and using the following formula:

$$\frac{\left(\left(sample\;-\;blank\right)\;-\;reference\right)}{reference}\cdot 100\%$$

To assess EV adsorption in the channels, EVs were labeled with SYTO™ RNASelect™ green fluorescent cell stain (Sigma) that selectively stains RNA according to the manufacturer’s protocol. Eight samples in total were prepared—four samples containing EVs with a concentration of 10^10^ particles/mL and four samples without EVs as a negative control; therefore, biological duplicates of EVs and negative control were analyzed for each single channel device type. Labeled EVs and negative control were washed with 45 mL of 0.02 µm filtered PBS and concentrated up to 200 μL by 100-kDa filters (Merck Millipore, MA, USA, #UFC910024) at 3000× *g* +4 °C for 60 min. Each sample is run through a separate prewashed single-channel device. Adsorption of EVs in the channels was visualized by laser scanning confocal microscopes (LEICA TCS SP8). An Argon (488 nm) laser was used to excite the SYTO™ RNASelect™ green fluorescent dye (excitation/emission, 490/530 nm). Images were obtained at a single focal plane with a 10x/NA 0.30 objective (Leica).

### 2.7. Bifurcated A4F Device Fabrication

**Figure 4:**
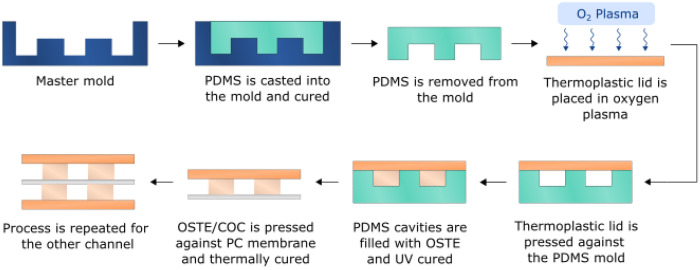
AF4 bifurcation EV separation device fabrication workflow. To retain membrane flatness during the assembly, the top piece was pre-bonded to the membrane before bonding the bottom piece.

To fabricate A4F bifurcation devices, two PDMS molds were used—one for the top channel (width 1 mm, height 0.5 mm, total length 210 mm) and one for the bottom channel (width 1.5 mm, height 0.6 mm, length 210 mm). The molds were prepared as described in the fabrication of OSTE-COC single-channel devices. Mixed OSTE was filled into the top channel PDMS mold sealed with COC luer slide as described above and cured with a UV dose of 850 mJ/cm^2^. The cured OSTE layer was brought in contact with a track-etched polycarbonate (PC) membrane (it4ip, Louvain-La-Neuve, Belgium) with a pore size of 50 nm and porosity of 11.8%. The assembly was cured overnight at 60 °C with an applied pressure of ~2 kPa. Following the same procedure, OSTE was filled into PDMS cavities sealed with a COC slide and cured with a UV dose of 1100 mJ/cm^2^. The cured OSTE was pressed against the PC membrane as described in Figure 4 and cured overnight at 60 °C with an applied pressure of ~1.6 kPa. The device bonding performance was tested using the protocol mentioned above.

### 2.8. Microfluidic Setup for Bifurcated A4F Experiments

**Figure 5:**
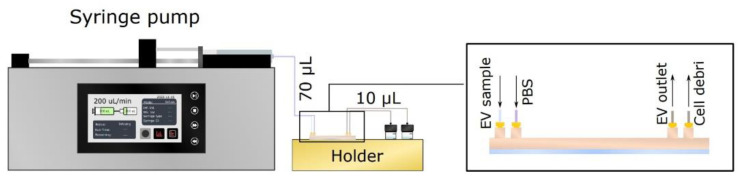
Syringe pump setup used to provide fluid flow for A4F bifurcation EV separation devices.

For the A4F bifurcation devices, a syringe pump system was used in order to ensure the continuous flow rate independent from pressure applied at various inlets. For this setup, the tubing principle was kept as for with the single channel devices—PTFE tubing for inlets and PEEK tubing for outlets to match resistance in outlets, as shown in Figure 5. For separation experiments, flow rates from 50 µL/min (25 µL/min for each inlet) to 1000 µL/min (500 µL/min for each inlet) were used.

### 2.9. Bifurcated A4F Experimental Setup and Sample Collection

Similar to single channel devices, the EV separation device was washed, left overnight, filled with 0.02 µm filtered PBS, and washed again the next day with buffer. Next, a bead mix sample consisting of two different size fluorescent beads—0.1 µm carboxylate FluoSpheres™ (Invitrogen, #F8803) and 1.0 µm polystyrene FluoSpheres™ (Invitrogen, #F13083) was prepared. The beads were uniformly dispersed in 0.02 µm filtered PBS with a concentration of 3.6 × 10^9^ beads/mL and 5 × 10^8^ beads/mL, respectively. A 5 mL syringe containing 1 mL of bead mix sample was attached to the EV inlet, and a 5 mL syringe containing 0.02 µm filtered PBS was connected to the PBS inlet (see Figure 5). Next, the syringe pump was started, and the outflow from each outlet was collected (EV and Cell debris outlet) in fractions of 200 µL. After the 1 mL bead mix sample had been collected, 1 mL of 0.02 µm filtered PBS was collected from all outlets as flowthrough to quantify if some of the beads were stuck into the device.

As a control, the same experiment was attempted with only the EV sample inlet. The inlet that was used for 0.02 µm filtered PBS was closed using mini-luer plugs and only the first inlet was connected to a syringe containing the bead mix sample. Outlets were collected and analyzed the same way.

Fluorescent beads were imaged using a confocal laser scanning microscope (TCS SP8, Leica, Germany). 8 µL of bead fraction was transferred to a glass slide, and a 22 × 22 mm cover slip was placed on top. Two lasers were used for the excitation of fluorescence-Argon (488 nm) was used for FluoSpheres™ carboxylate, 0.1 µm yellow-green (Excitation/emission, 505/515) (Invitrogen), and DPSS (561 nm) was used for FluoSpheres™ polystyrene, 1.0 µm, red (580/605) (Invitrogen). Images were obtained at a single focal plane with 63x/NA 0.70 objective (Leica).

### 2.10. EV Isolation from Cell Media by Microfluidic Device in Comparison to SEC

To test the developed EV device for EV isolation, 5 mL of unconditioned ASC52telo cell line media without serum, as described in the Section 2.1, was spiked with 5 × 10^9^ particles/mL. Both EV spiked media and PBS buffer were administrated through inlets at 250 μL/min and collected at the EV outlet and cell debris outlet. Collected liquids at each outlet were concentrated by up to 250 μL using 3 kDa filters at 14,000× *g* for 2 h at +4 °C, followed by NTA measurements. At the same time, 5 mL of spiked EV media was concentrated up to 500 μL using a 100 kDa filter and used for SEC by using Izon qEVoriginal/35 nm columns (Izon, Christchurch, New Zealand #SP7), similarly as previously described in Section 2.1, concentrated by up to 200 μL using 3 kDa filters afterwards and analyzed by NTA. Both experiments with SEC and OSTE-COC devices were performed in biological duplicates.

### 2.11. Direct EV Isolation from Cell Media Using UC, SEC and Device

ASC52telo cells were cultured as explained before in Section 2.1 till cells were transferred to the bioreactor. Cell media was collected and centrifuged at 300× *g* for 5 min room temperature, followed by 30 min at 10,000× *g*, centrifugation at 4 °C in order to remove cells and apoptotic bodies. Then, 20 mL of supernatants were used to isolate EVs by three different mechanisms: ultracentrifugation (UC), SEC, and using our device. Ultracentrifugation was performed as follows: supernatant was centrifuged twice at 100,000× *g* for 70 min using a Ti70 rotor (Beckham Coulter Life Sciences, IN, USA) followed by 70 min at 100,000× *g* in an SW40 rotor (Beckham Coulter Life Sciences, IN, USA) prior pellet resuspension in previously filtered 0.02 µm PBS. All the procedure was done at 4 °C. SEC procedure was executed as explained in Section 2.1, and the EV isolation technique using our device was performed following the instructions detailed in Section 2.10 without the initial spiking EVs step.

### 2.12. Image and Data Analysis

Data were analyzed, and graphs were made by GraphPad Prisma 8.0. Statistical significance between two groups was calculated by the Mann-Whitney U test, and the Friedmans test with Dunn’s correction was used when more than 2 groups were assessed. *P*-values below 0.05 were considered significant. Bead counting was done in ImageJ 1.53 t software via the following algorithm. Images were first converted to 8 bits for thresholding, where the value was set to 0.1%, and after that Analyze Particle module was used. Bead analysis was done in MS Excel, and the graphs were made using Origin Pro 9.0.

## 3. Results

### 3.1. EV Isolation, Quantification, Characterizations

**Figure 6:**
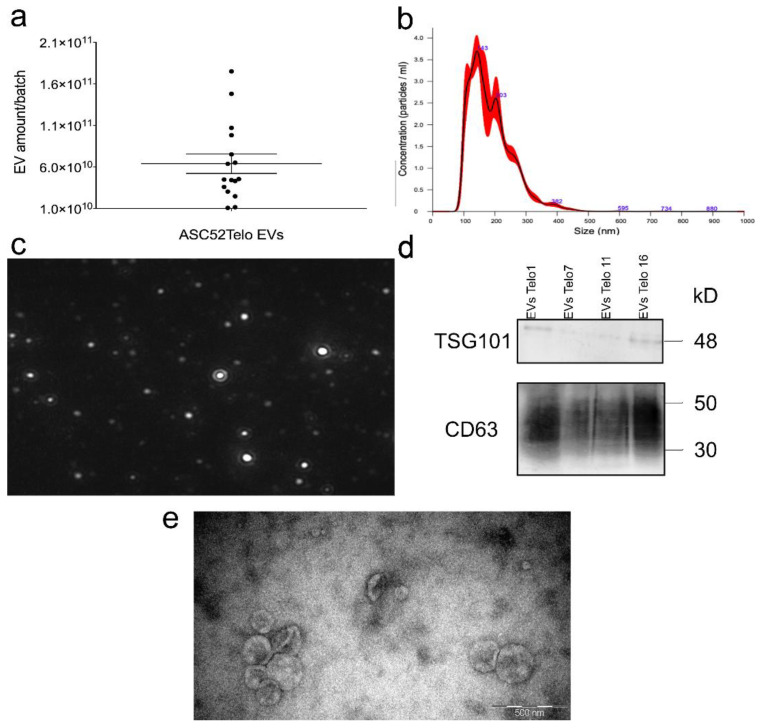
ASC52Telo EVs characterization. (**a**) Total amount of ASC52Telo EVs recovered per bioreactor batch with the corresponding mean and SEM (**b**) Representative picture of diluted ASC52 Telo EVs size distribution. (**c**) Image of EVs as seen in Nanosight (**d**) Western Blot of TSG101 and CD63 expression in EVs from 4 different ASC52Telo EVs batches (**e**) Representative TEM image of ASC52Telo EVs.

To test the efficacy of the device in isolating EVs, we cultured and isolated EVs from 16 batches of ASC52Telo cell bioreactor, as explained before. We chose to work with mesenchymal stem cell-derived EVs due to their therapeutical potential [30] since the idea was to mimic the use of our device in a real-case scenario. In addition, we chose to culture them in a bioreactor since this provided the best medium for growth in a scalable manner with minimum disturbance [31], allowing the collection of high-yield EVs. Recovered ASC52Telo EVs number, size, and purity were characterized by NTA, TEM, and WB. Results can be seen in Figure 6. Data showed that the total amount of EVs recovered per batch ranged from 1.08 × 10^10^ to 1.75 × 10^11^, with an average of 6.39 × 10^10^ (see Figure 6a), and EVs ranged in size from 80 nm to 480 nm; with a mean of 187 nm +/− 2 nm and a mode of 142 nm +/− 9 nm (see Figure 6b) based on NTA image analysis (Figure 6c) that correlated with TEM results (see Figure 6e). WB data revealed positive results for CD63 and TSG101, both positive markers for EVs that can be seen in Figure 6d. This data correlates with the expected EV phenotype and size [25]; thus, proving we successfully isolated EVs from ASC52Telo cell cultures for further experiments.

### 3.2. Material Selection for Microfluidic Devices

**Figure 7:**
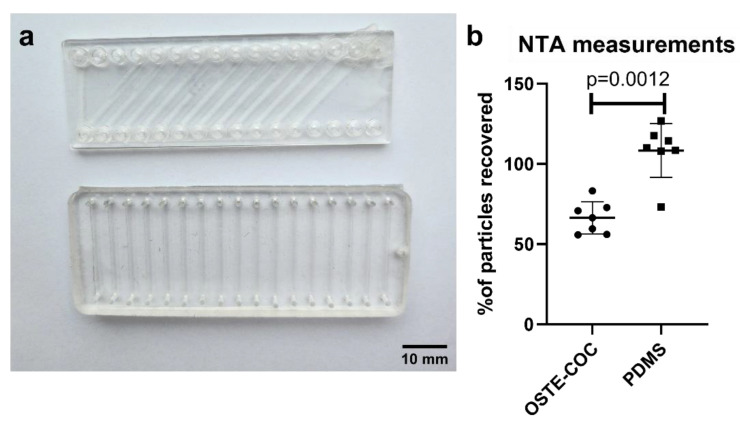
Single channel experiments for EV-loss quantification. (**a**) Top-OSTE—COC single-channel devices. Bottom-PDMS single channel devices. Both devices have the same microfluidic channel dimensions, but the COC device has a slanted channel layout to accommodate this length on a microscope slide format, (**b**) Scatter dot plot of EV recovery percentage from OSTE-COC and PDMS single channel devices with mean and SD.

Single channel testing devices were fabricated in a cleanroom environment with equal channel dimensions for both OSTE-COC and PDMS devices, which can be seen in Figure 7a, respectively.

Next, to fully characterize the microfluidic device effect on EV recovery, we compared PDMS single-channel devices with OSTE-COC polymer single-channel devices (previously shown as superior materials to PDMS in terms of small molecule adsorption for use cases in organ on the chip field) [16]. In these experiments, we used previously selected pressure as optimal-100 mBar. A pressure system was applied since this allows the use of low binding 1.5 mL Eppendorf tubes to reduce any EV adsorption into the system. EV dilutions were made in the upper working range of the NTA (5 × 10^8^ particles/mL) since part of EVs can be adsorbed in devices. Before experiments, EV dilutions were measured again with NTA, since particle dilutions can vary from calculated based on particle nature and NTA measurement technology. Seven biological replicates were performed since there was expected variation between replicates based on particle nature in different EV batches, device fabrication batches and NTA technology, which is well known [32]. The average recovery of EVs from OSTE-COC was 66.3% while variability between replicates was up to 27.3%. EV recovery from PDMS average was 108.3% with variability between replicates up to 53.6% (see Figure 7c). To ensure no EV adsorption and release take place, all microfluidic channels were used only once. While variation between replicates may seem huge, this is an expected result in comparison to standard EV isolation method efficacy, where EV recovery can vary up to 90% [33,34,35,36]. OSTE-COC devices showed lower variation than PDMS, while in flowthrough from PDMS devices particles, were constantly higher than in the reference sample, suggesting significant particle leakage from PDMS that cannot be detected in blank measurements due to the total amount being out of the NTA working range. Differences between devices were statistically significant, suggesting that OSTE-COC outperforms in reproducibility PDMS and does not produce additional particles in the expected EV size range. However, to prove that the increased particle amount is due to the PDMS leakage, additional experiments on the chemical composition of EV flowthrough are necessary. It is likely that these are PDMS particles from the aggregation of uncured monomers since it has been reported that PDMS tends to leach monomers [37].

To test if the decreased recovery of EVs is due to the adsorption, we performed EV staining by Sytox, which is widely used in EV staining [38]. Endogenic dye was used since PDMS is well known to absorb also lipophilic dyes, such as EVs membrane dyes that can create additional bias in comparison; therefore, EV nucleic acid enclosed staining was selected. There was no EV adsorption detected nether in OSTE-COC nor PDMS devices (data not showed), suggesting that the method of choice is not sensitive enough or EV loss is due to the EV bursting; thus, cargo is spilled, which requires additional analysis of lipid amount in flowthrough [16].

### 3.3. Asymmetric Field Flow Fractionation Experiments with Model System

**Figure 8:**
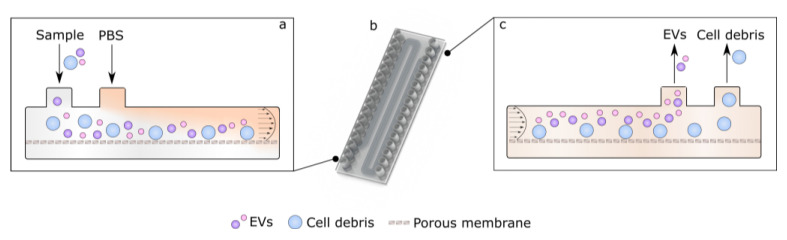
The principle of operation for bifurcated A4F method is described here. (**a**) Start of the device with sample and buffer inlets. (**b**) CAD image of the device showing meandering nature of channels for increased interaction length. (**c**) End of the device with outlets showing the separated result of EVs and large debris.

Field-flow fractionation (FFF) is a method for entity separation laterally in a channel by exerting an external force onto the particles. A4F utilizes a liquid permeable membrane at the bottom of the channel; thus, as the fluid moves through the channel, a downward force is exerted onto particles in the channel. The equilibrium position of the particle laterally is determined by the sorting force, which acts downwards, and particle diffusion, which produces an effectively upwards-pointed force. Thus, particles with different diffusion constants (dependent on various parameters, but of interest here—size) are separated. Herein, a novel design to achieve EV separation using A4F is proposed, as seen in Figure 8. To reduce the separation time of the EVs and force the buffer exchange, an EV-containing solution and a buffer PBS are flown into a bifurcation design as per Figure 8a. This bifurcation forces EVs closer to the porous membrane spatially during the start of separation, allowing for shorter separation times as opposed to standard A4F methods (see meandering nature of channels in Figure 8b), where EVs are only focused from sides, thus leading to longer times for separation due to EV distribution across the whole channel height (see Figure 8c) [20,39]. This also forces buffer exchange as the crossflow will eventually remove all the original EV-containing sample buffer, thus leading to a reduction of protein and small molecule contamination within the EV sample. Poiseuille flow then physically separates the particles, which are collected at bifurcated outlets according to size fraction.

**Figure 9:**
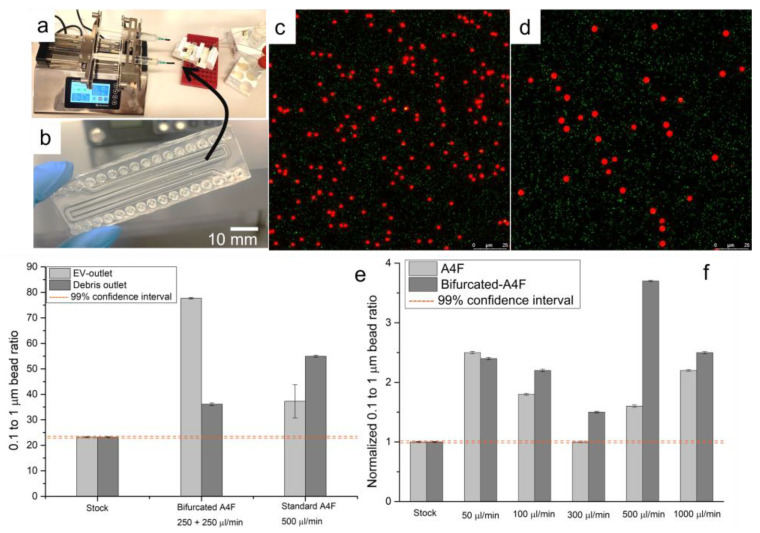
Bifurcated A4F experiments with control particles. (**a**) Experimental setup with a syringe pump and resistances in the ports to equalize hydraulic resistances of outlets. (**b**) Fabricated A4F device with meandering design for increased total length in the channels. (**c**) A sample image of overlaid red and green channels for 1 µm and 100 nm bead from confocal microscopy, image from the stock prior to experiments. (**d**) A sample image of overlaid red and green channels for 1 µm and 100 nm bead from confocal microscopy, image from bifurcated A4F experiment with 1 mL/min flow rate. In the bifurcated A4F beads experience 1 in 2 dilutions with respect to stock (**e**) Ratio of 100 nm to 1000 nm beads in both particle outlets of the chip for bifurcated A4F and standard A4F experimental setup. (**f**) A sweep of flow rates for bifurcated and standard A4F setup with stock-normalized 100 nm bead plot.

The experimental setup of bifurcated A4F can be seen in Figure 9a. Fabricated devices can be seen in Figure 9b, where the top and bottom channels are separated by the track-etched membrane, and both channels follow the meandering channel path. The bonding performance was also tested by passing filtered DI water through at 1 mL/min. To evaluate the proof of concept of the devices, a mix of polystyrene beads with 100 nm and 1000 nm diameters were made, which size-wise would represent vesicles [1] and fine cell debris [40]. The pre-made mix was used throughout the bead quantification experiments and had a small to large bead ratio of 23.2 ± 0.26 quantified via image analysis of confocal microscopy (see Figure 9c for exemplary stock bead mix). Experiments were done with varied flowrates for the bead mix with and without the bifurcating flow to investigate the linear velocity effect on the separation efficiency. Given the key outreach of the system is the recovery of samples via the outlets of interest, it is important to understand the quantification of the samples. If there is an increase of 100 nm bead concentration (which represents EVs) in the EV outlet of Figure 8b, this would be read as an increase in the small to large bead ratio (i.e., shift towards larger ratios than the stock 23.2 to 1). Similarly, an increase in large bead concentration (the cell debris model system) in the debris outlet would be represented by a decrease in the small to large bead ratio (i.e., shift smaller ratios than the starting 23.2 to 1).

Experiments were done with and without the bifurcating flow, which served as a controlled study, thus allowing us to evaluate the effect of bifurcating buffer on the particle separation. The bifurcating to sample flow rate was fixed at a 1:1 ratio, but this could be altered to selectively adjust the separated particle size from a more dispersed size population sample. Figure 9d,e shows a confocal sample image and graph of the bead separation with a 1:1 ratio and the total of 500 µL/min flow rate in the channels, respectively. The bifurcated A4F leads to significant increase in the small bead ratio in the EV-outlet, whereas in the standard A4F setup, the separation is not as pronounced. The elevated small particle ratio in the debris outlet is also worth noting. Throughout the experiments, small bead recovery stays over 90%, whereas the large bead recovery is in the 40–50% range, which is due to the high sedimentation experienced by the beads, which takes place in the long channels.

Due to sedimentation observed by large particles, subsequent flow-rate analysis has been done comparing the extracted particles from small particle outlets with respect to the stock solution. A sweep of various total flow rates for the bifurcating and regular A4F designs was done in the range from 50 µL/min to 1000 µL/min (e.g., 50 µL/min means that both sample and buffer solutions were introduced in the respective inlets at 25 µL/min) to evaluate the performance of the experimental setup. Figure 9f shows the normalized bead recovery with respect to stock dependence on the flow rate. It is worth noting the below one recovery on the lower flow rates that indicate the loss of small particles in the system without the increase in concentration. Bifurcated A4F setup outperforms standard setup at higher flow rates, especially as flow rates exceed 100 µL/min.

### 3.4. Initial Experiments with Bioreactor-Grown Extracellular Vesicle Separation

Further, to show the proof of principle for EV separation, bioreactor-grown EVs were separated from cell culture media as described in Section 2.1. Following EV size characterization according to MISEV2018 standards. Next, EVs were spiked in non-conditioned bioreactor media, and the bifurcation A4F experiments were repeated with 250 µL/min EV media sample and 250 µL/min PBS buffer flow rates based on bead experiments. Unconditioned bioreactor media was selected since the liquid properties mimic those where this kind of device could be used in the future to isolate therapeutic EVs. Also, unconditioned media was important for this experiment to avoid any EVs that come from animal compounds or cell sources, subsequently allowing a spike in a certain amount of well-characterized EVs to evaluate EV recovery and size distribution and compare results with one of the gold standard methods in EV isolation SEC.

**Figure 10:**
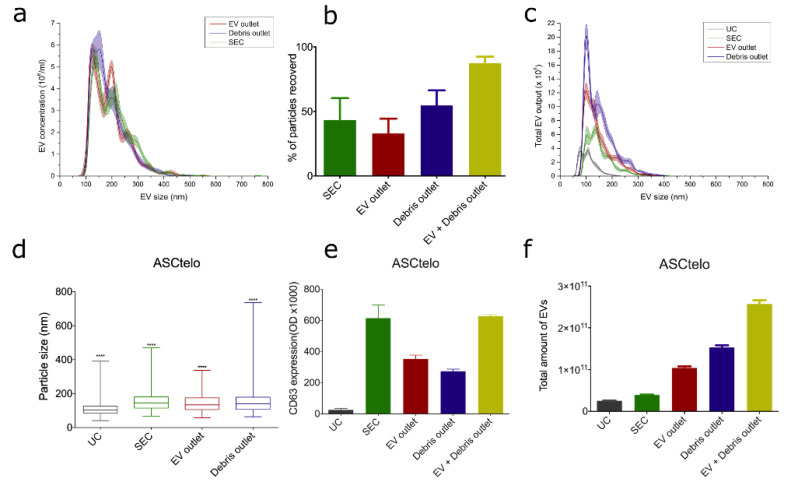
(**a**) Size distribution of particles per ml from EV outlet and debris outlet. Lines mean average value, while shaded area shows SD in measurements. (**b**) Percentage recovered of EV from EV spiked unconditioned bioreactor media by SEC and OSTE-COC device. Bars represent mean while whiskers SD. (**c**) Size distribution plot of total EVs isolated from ASC52-telo conditioned media by SEC, UC, EV outlet and Debris outlet of OSTE-COC device. (**d**) BoxPlot of sample size distribution obtained from ASC52telo cell media using different EV isolation techniques. **** = *p* < 0.0001. (**e**) CD63 ELISA expression in mean +/− SEM. (**f**) Total number of recovered particles from ASCtelo cells media using different isolation techniques in mean +/− SD.

Figure 10a,b shows the EV population distribution changes and EV recovery from EV spiked media by using SEC and A4F devices from OSTE-COC. Figure 10a shows the EV-size distribution for samples (from Figure 6b) flown into the bifurcated A4F device and SEC. There is a slight shift of 200 nm particle size towards the EV-inlet, which is not present in the debris outlet anymore. However, there was no statistical significance detected in size distribution between SEC, EV outlet, and debris outlet, which can be explained by the homogenous EV sample spiked into cell media as described in Figure 6. Notably, the 100 nm peak for both outlets stayed within the measurement error range. This can be explained by the fact that cross-flow induced force is particle volume dependent; subsequently, 100 nm EVs experience significantly smaller force. Furthermore, 100 nm EVs have a significantly smaller buoyance compared to similarly sized polystyrene beads, which were used in parameter sweep [41].

EV recovery experiments showed that OSTE-COC devices have better results than SEC in terms of EV recovery (see Figure 10b) if the EV amount of both outlets are combined; separately, the results were similar to SEC. Based on EV size distribution measurements current device with applied flowrates cannot collect all EV-size particles at the EV outlet and separate them from the rest. This can be explained by volume flow rates selected from bead experiments that are not optimal for EV experiments and would have to be optimized for each media composition. Additionally, unconditioned media can play a role in necessary flow rates since it can differ in liquid properties in comparison to PBS used in bead experiments and buffer. The viscosity of liquid has a direct effect on the downward force of the particles, with more viscous media yielding worse particle separation. This suggests a necessity in the future for the sample and buffer flow optimization before experimenting with large-volume samples. While this is not a serious concern for bioreactor samples, where conditions are kept uniform, this can prove challenging for heterogenic high-volume samples such as urine. Next, to test the device with more heterogenic EV samples-ASC52-telo cell conditioned media from flask cultures was selected for EV isolation and comparison between SEC, UC, and A4F devices. Results showed that in more heterogenic particle samples A4F device EV outlet statistically significantly outperforms UC, SEC, and Debris outlet in terms of particle size distribution and homogeneity (see Figure 10c,d). A considerable proportion of EVs was also detected in the Debris outlet channel based on CD63 dsELISA tests (see Figure 10e), however statistically significantly increased number of larger particles (>400 nm) in the Debris outlet channel (see Figure 10d) confirms of A4F device ability separate EVs from larger particles. To improve particle collection in the size of small EVs (100–200 nm), EVs outlet sample and buffer flow rates optimization in the future is necessary. Based on results in Figure 10f, both the EV outlet and Debris outlet have more particles than SEC and UC, while dsELISA shows (Figure 10e) that SEC contains CD63 signal similar to EV and Debris outlet combination, therefore demonstrating that the A4F device collects all the same particles as SEC combining EV and Debris outlet. The rest of the particles can be EVs without the CD63 marker at least in EV outlets that were lost during SEC since only part of EVs contains CD63 markers and generally there is no single, unique marker for EVs [25]. While in the Debris outlet, larger particle identity is unclear since these can be large EVs or cell debris. To find out the particle nature, especially in Debris outlets that are not CD63 positive, additional particle analyses are necessary, such as through high-resolution imaging flow cytometry [42]. However, this is beyond the scope of this article.

Nevertheless, these results show the potential to utilize PDMS—free bifurcated A4F device design for continuous EV separation from larger entities. This method has potentially smaller EV-retention or loss within the devices compared to the golden standard in the form of SEC.

## 4. Discussion

We have already previously discussed the importance of microfluidic material selection for application within the organ on the chip field [16]. Arguably, within the EV field, material properties play an even higher role, given the small scale and nature of the EVs, which is exacerbated by the fact that no EV quantification method provides absolute clarity on the true amount of EVs present [32]. Subsequently, there is a high need for the reduction of material-specific effects in microfluidic devices. As reported herein, the PDMS has high non-specific particle release even after thorough washing, which cannot be detected before since NTA has detection limits but apparently affects final measurements, which inevitably can cause downstream problems for EV quantification and functional characterization [25]. Therefore, we believe that reported study of OSTE-COC devices has a promising potential to eventually aid the replacement of PDMS as the standard material of choice for EV-related devices.

Herein shown bifurcated A4F setup for nanoparticle and EV separation lies under the label-free, passive, size-based separation classification introduced by de Mello group [43]. Subsequently, the method should be evaluated in terms of performance with respect to deterministic lateral displacement (DLD), viscoelasticity-based and filtration-based separation of the EVs. Both DLD and viscoelasticity-based separation methods excel in terms of absolute EV purity, with purities of around 90% reported [44,45,46]. However, the flow rate and subsequent throughput of both systems are usually low due to the small forces involved, with both DLD and viscoelastic systems reporting flow rates of 1–2 mL/h. As a direct comparison, the lowest reported flow rates herein start from 3 mL/h and go as much as 60 mL/h, with 3.7X enrichment of small beads at 30 mL/h flow rates. Regarding EV extraction purity using a bifurcated A4F setup, an enrichment of 3.7 times over the initial population distribution can be seen, highlighting the promising potential of this technique. Furthermore, in terms of absolute values, more than 90% of the 100 nm beads are recovered from the devices, whereas smaller recovery is seen for debris-sized beads suggesting the likely single-use nature of enrichment devices with a design-dependent capacity for debris collection.

Due to the nature of the fabrication protocol described here, upscaling device fabrication to account for the single-use nature would not be impossible, and reaction injection molding principles have already been outlined [47]. This would also aid with the repeatability issues typically found in PDMS devices.

Briefly, the reaction injection molding process is reliant on a continuous flow two-part mixing of polymers, which is injected into the mold at lower pressure before triggering the polymerization [48]. In the case of OSTE devices, to maintain high throughput, master molds would need to be fabricated from polished steel or aluminum to dissipate the exothermic reaction of OSTE polymerization. [44]. Furthermore, polished steel mold inserts would be able to ensure sufficient contact between COC thermoplastic pieces and mold to prevent OSTE ingress under the channel regions. The 3D printed mold surface roughness herein was compensated via the use of flexible master mold material. Subsequently, with the use of heat-dissipating molds and reaction injection molding setup, the overall cycle time per injection shot could be reduced to a few minutes at most, leading to significantly higher fabrication throughputs.

Both PDMS-free devices and a focus on high-throughput systems underscore the applicability of herein presented devices for the EV industry, which has been growing exponentially. Furthermore, the need to switch to higher sample volumes and EV amount has been highlighted previously if EVs are to make a significant impact within many clinical trials reported, as well as the novel applications in various industries, such as cosmetics [49].

Subsequent studies should be devoted towards EV separation from biologically relevant cell culture media and human samples, such as urine, where the currently utilized methods struggle with the vast quantity of the biological media for processing by optimizing different flow rates of both sample and buffer. Furthermore, future EV-related quantitative and qualitative evaluation of the separated populations should be done according to MISEV standards, which subsequently should include transmission electron microscopy, NTA, and other requirements which are beyond the scope of this study [25].

Finally, it is worth noting that equally to DLD and viscoelastic-based separation, herein shown bifurcated A4F separation does not do sample concentration; instead, specific particle purity is increased. Subsequently, a dedicated sample concentration module for further sample processing might be required for high-volume samples after EV separation. For instance, the ExoTIC device by Liu et al. or simple centrifugation in concentration tubes [50,51].

## 5. Conclusions

In conclusion, our manuscript presented a novel method for separating extracellular vesicles from large sample volumes using bifurcated asymmetric field flow fractionation in PDMS-free microfluidic devices. We demonstrated the effectiveness of this method through the separation of extracellular vesicles from large sample volumes, as well as the analysis of microfluidic system parameters using polystyrene beads. With a control system in the form of beads, we could reach 3.7 times enrichment over stock, whereas EV samples showed clear population distribution changes in the 200 nm range.

The use of PDMS-free microfluidic devices allows to a scalable and efficient approach for the separation of extracellular vesicles, addressing the scalability issues commonly encountered with PDMS-based materials. Overall, this research contributes to the growing body of knowledge on extracellular vesicle separation and the potential applications of microfluidic technology in this area.

## Data Availability

Not applicable.
